# Supplementary material for: Enhanced Thermal Stability of Carbonyl Iron Nanocrystalline Microwave Absorbents by Pinning Grain Boundaries with SiBaFe Alloy Nanoparticles
Source: Nanomaterials (Basel). 2024 May 16;14(10):869. doi: 10.3390/nano14100869 (PMC11124353; doi:10.3390/nano14100869)
Supplement: Supplementary file 1 [file nanomaterials-14-00869-s001.zip › nanomaterials-2942926-supplementary.pdf]

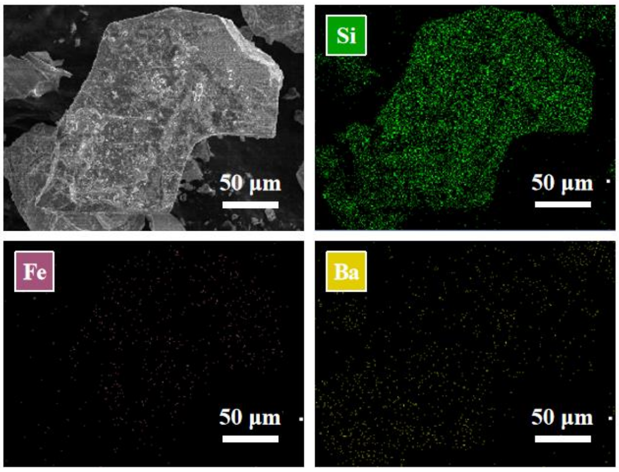

Figure S1. EDS images of raw SiBaFe alloy particles.

Table S1. Composition (except C and O) of raw SiBaFe alloy particles.

**Elements**	Si	Ba	Fe	Ca
**Contents (wt.%)**	50.93 ± 4.04	28.98 ± 3.44	17.40 ± 2.31	2.69 ± 1.31

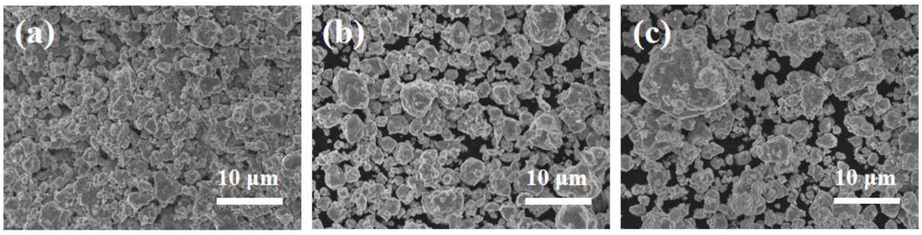

Figure S2. (a-c) SEM images of **(a)** CI, **(b)** CI/SiBaFe-5 and **(c)** CI/1SiBaFe-10 particles.

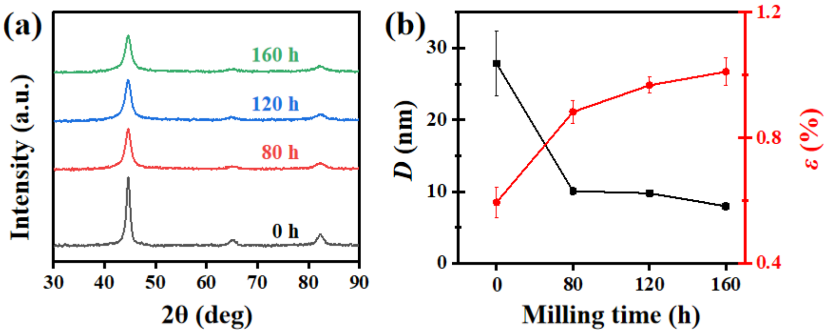

Figure S3. (a) XRD spectra, **(b)** grain size and internal strain of CI/SiBaFe-15 particles before and after milling for different times.

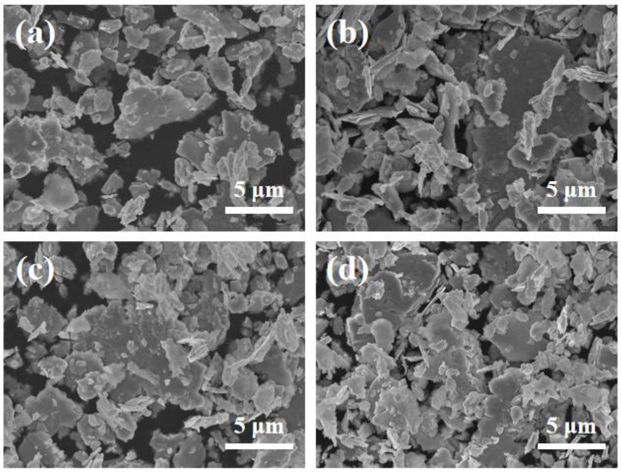

Figure S4. (a-d) SEM images of flaky **(a)** CI, **(b)** CI/SiBaFe-5, **(c)** CI/1SiBaFe-10 and **(d)** CI/1SiBaFe-15 particles after attritor ball milling.

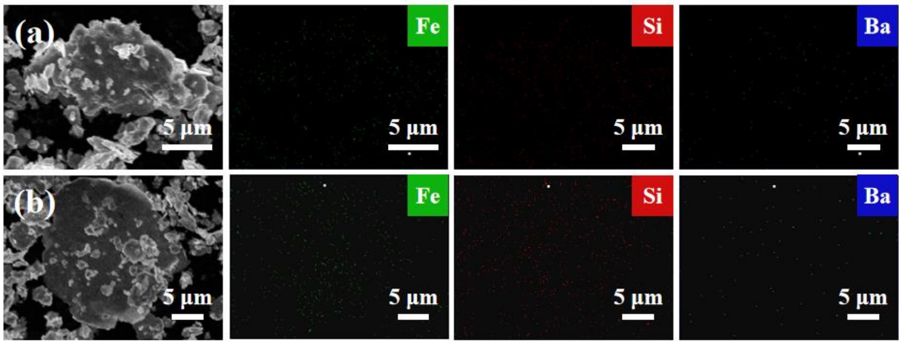

Figure S5. (a,b) EDS images of **(a)** flaky CI/SiBaFe-5 and **(b)** CI/SiBaFe-10 particles.

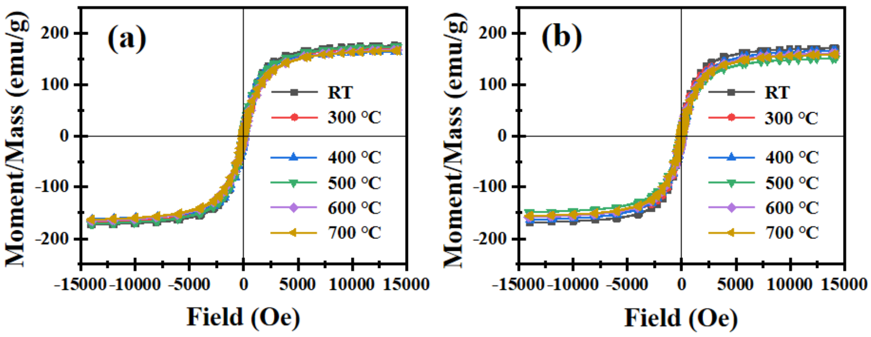

Figure S6. Hysteresis loops of flaky **(a)** CI/SiBaFe-5 and **(b)** CI/SiBaFe-10 particles before and after heat treatment.

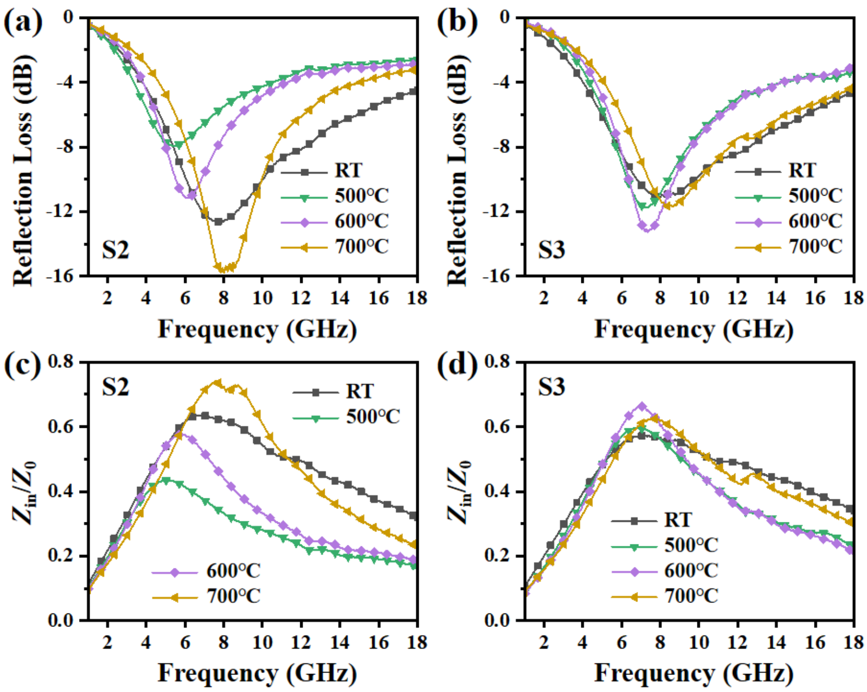

Figure S7. (a,b) Microwave absorption of **(a)** CI/SiBaFe-5 and **(b)** CI/SiBaFe-10 particles; **(c,d)** impedance matching analysis for **(c)** CI/SiBaFe-5 and **(d)** CI/SiBaFe-10 particles before and after heat treatment.
